# Cold Preflush of Porcine Kidney Grafts Prior to Normothermic Machine Perfusion Aggravates Ischemia Reperfusion Injury

**DOI:** 10.1038/s41598-019-50101-7

**Published:** 2019-09-25

**Authors:** Gregor Fabry, Benedict M. Doorschodt, Tim Grzanna, Peter Boor, Aaron Elliott, André Stollenwerk, René H. Tolba, Rolf Rossaint, Christian Bleilevens

**Affiliations:** 1 Department of Anesthesiology, Medical Faculty, RWTH Aachen University, Aachen, Germany; 2 Department of Intensive Care and Intermediate Care, Medical Faculty, RWTH Aachen University, Aachen, Germany; 3 Institute for Laboratory Animal Science & Experimental Surgery, Medical Faculty, RWTH Aachen, Aachen, Germany; 4 Department of Thoracic and Cardiovascular Surgery, Medical Faculty, RWTH Aachen, Aachen, Germany; 5 Institute of Pathology & Division of Nephrology, Medical Faculty, RWTH Aachen, Aachen, Germany; 6 Informatik 11-Embedded Software, RWTH Aachen University, Aachen, Germany

**Keywords:** Preclinical research, Organ transplantation, Experimental models of disease, Kidney

## Abstract

Normothermic machine perfusion (NMP) of kidney grafts is a promising new preservation method to improve graft quality and clinical outcome. Routinely, kidneys are washed out of blood remnants and cooled using organ preservation solutions prior to NMP. Here we assessed the effect of cold preflush compared to direct NMP. After 30 min of warm ischemia, porcine kidneys were either preflushed with cold histidine-tryptophan-ketoglutarate solution (PFNMP group) prior to NMP or directly subjected to NMP (DNMP group) using a blood/buffer solution. NMP was performed at a perfusion pressure of 75 mmHg for 6 h. Functional parameters were assessed as well as histopathological and biochemical analyses. Renal function as expressed by creatinine clearance, fractional excretion of sodium and total output of urine was inferior in PFNMP. Urine protein and neutrophil gelatinase-associated lipocalin (NGAL) concentrations as markers for kidney damage were significantly higher in the PFNMP group. Additionally, increased osmotic nephropathy was found after PFNMP. This study demonstrated that cold preflush prior to NMP aggravates ischemia reperfusion injury in comparison to direct NMP of warm ischemia-damaged kidney grafts. With increasing use of NMP systems for kidneys and other organs, further research into graft flushing during retrieval is warranted.

## Introduction

In kidney transplantation, the most widely employed method for graft preservation is the use of a cold preflush to remove blood remnants followed by cold storage (CS) at 4 °C, using a preservation solution such as histidine-tryptophan-ketoglutarate (HTK), University of Wisconsin solution (UW), Celsior or others^1^. Hypothermic machine perfusion (HMP) of the kidney is increasingly employed for preservation of kidney grafts, either additionally after CS^2^ or as a replacement of CS^3^. HMP and, to a greater extent, normothermic machine perfusion (NMP) appear to be superior as preservation method when compared to CS^4,5^, potentially increasing the donor pool by improving the outcome of transplantation of grafts from extended criteria donors (ECD) as well as from donation after cardiac death (DCD)^6^. Kidney grafts preserved by NMP sustain less ischemic reperfusion injury after warm ischemia, even when compared to immediately transplanted non-stored kidneys^7^. Also, graft quality assessment during NMP has recently enabled successful transplantation of declined kidneys^8,9^. The optimal perfusion conditions for renal NMP have still to be determined. However, some evidence suggests that normothermic pressure-controlled carbogen-ventilated perfusion is favorable^10–12^. The reported perfusion duration appears to be limited in renal NMP, since most studies describe up to 6 h of perfusion time, while only single studies report up to 24 h^3,13^, a duration that is already routinely achieved in clinical trials of liver NMP^14^.

To our knowledge, in recent NMP studies, even in those without a prior CS period, kidneys were preflushed with cold preservation solutions, either separately or during systemic washout of the abdominal organs before organ retrieval. These solutions were designed to provide optimal CS preservation conditions over several hours, not only for flushing. In order to further improve kidney graft quality, the effects of cold preflush should be further examined in the context of NMP. The only study describing direct machine perfusion without cold preflush reported greater ischemia reperfusion injury in kidneys flushed with cold preservation solution followed by CS than in the direct perfusion group^15^. The results indicated a trend towards lowered kidney function in the cold flush and storage group, however the NMP time was limited to 145 min.

In this study, we aimed to investigate the isolated effect of cold preflush without subsequent cold storage. We hypothesized that cold preflush before normothermic perfusion (PFNMP) aggravates kidney graft injury in comparison to normothermic perfusion directly after retrieval (DNMP). We aimed to compare the extent of ischemia reperfusion injury between both groups during 6 h of NMP.

## Material and Methods

### Experimental protocols

The experimental protocol was approved by the Institutional Animal Care and Use Committee of the RWTH Aachen University Hospital and performed in accordance with German legislation governing animal studies following the ‘*Guide for the care and use of Laboratory Animals’* (NIH publication, 8^th^ edition, 2011) and the Directive 2010/63/EU on the protection of animals used for scientific purposes (Official Journal of the European Union, 2010).

**Figure 1:**
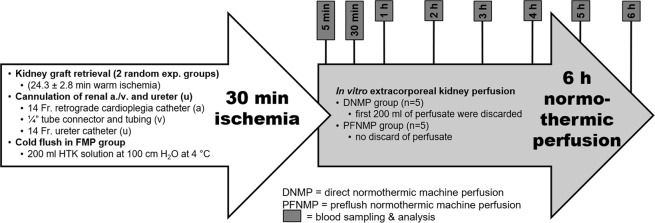
Schematic overview of experimental design.

After retrieval and cannulation of both kidneys from the same animal, prior to normothermic perfusion for 6 h, one kidney was preflushed with HTK solution (PFNMP group, n = 5), whereas the other kidney was not preflushed (DNMP group, n = 5). Perfusate and urine samples were collected, and operational parameters were recorded at eight points in time (Fig. 1). After 6 h, tissue samples were collected for histological staining and western-blot analyses.

### Kidney retrieval

Female German Landrace pigs with 55,2 ± 1,9 kg body weight (BW, mean ± SEM) from a disease-free barrier breeding facility were housed in fully air-conditioned rooms (22 °C room temperature, 50% relative humidity) and allowed to acclimatize to their surroundings for a minimum of seven days and fasted for 12 h before surgery with free access to water. The animals were premedicated with 8 mg/kg BW azaperone (Stresnil, Janssen-Cilag GmbH, Neuss, Germany), 15 mg/kg BW ketamine (Ceva GmbH, Duesseldorf, Germany) and 10 mg atropine (1 ml/1% atropine sulfate, Dr. Franz Köhler Chemie GmbH, Bensheim, Germany) administrated intramuscularly. After cannulation of the femoral vein, 600 mL of venous blood for the *ex vivo* perfusion of the kidneys was withdrawn into sterile blood bags filled with 5,000 IU of heparin each (B. Braun Melsungen AG, Melsungen, Germany). The animals were thereafter euthanized by an IV administration of 1 mL/kg BW pentobarbital (Narcoren, Merial GmbH, Hallbergmoss, Germany). After cardiac arrest, a midline laparotomy was performed and both kidneys were explanted simultaneously to achieve an equal warm ischemic time. The duration from cardiac arrest until explantation was recorded and regarded as warm ischemic time. In compliance with the 3R principle, other organs of the animals were retrieved for different in-house research purposes.

### Perfusion preparation

After organ explantation, the kidneys were weighed, and the renal artery was cannulated (retrograde cardioplegia catheter, 14 Fr., Edwards Life Sciences; Unterschleißheim, Germany) as well as the renal vein (¼” tube connector, ¼” tubing, free life medical GmbH, Aachen, Germany) and ureter (14 Fr. catheter; Convatec (Germany) GmbH, Munich, Germany). In the PFNMP group, flushing of the kidney was performed through the renal artery with 200 ml HTK solution (Dr. Franz Köhler Chemie GmbH, Bensheim, Germany) added with 5,000 IU of heparin at 4 °C at a hydrostatic pressure of 100 cmH_2_O. The kidneys in the DNMP group were cannulated without flushing. Thereafter, in both groups the kidneys were connected to the NMP system and perfusion was started simultaneously at 38 °C for 6 h. In the DNMP group, the first 200 ml of perfusate out of the venous catheter were collected and discarded.

### Perfusion medium

As perfusion medium, a Ringer’s-based solution adapted from established NMP protocols^16,17^ mixed with leucocyte-depleted whole blood was used (Table 1). Mannitol (5 g/l, B. Braun Melsungen AG, Melsungen, Germany), sodium hydrogen carbonate (2.1 g/l, Sigma-Aldrich Chemie GmbH, Steinheim, Germany), creatinine (0.116 g/l, Sigma-Aldrich Chemie GmbH), glucose (1.98 g/l, Sigma-Aldrich Chemie GmbH) and heparin (5,000 IU/l, B. Braun Melsungen AG) were dissolved in 600 ml of Ringer’s solution (B. Braun Melsungen AG). The perfusion circuits were primed with 350 ml of the Ringer’s-based solution in the DNMP and 250 ml in the PFNMP group. The autologous blood was leucocyte-depleted by a standard filter and 350 ml was added to the DNMP and 250 ml to the PFNMP group. This resulted in an excess of 200 ml of perfusate in the DNMP group, which matched the amount of perfusate discarded after perfusion start.

**Table 1:** Composition of perfusate for each circuit.

Perfusate
250 ml Ringer’s solution with added:
-Mannitol 5 g/L
-Sodium bicarbonate 2,1 g/L
-Creatinine 0,116 g/L
-Glucose 1,98 g/L
250 ml leucocyte-depleted autologous whole blood
*Osmolarity: 290 mosm/L*, *Oncotic pressure: estim*. *8*.*5 mmHg*
**Urine replacement**
Ringer’s solution
**Nutritive solution**, **added at 11**,**2 ml/h**
Nutriflex peri 44 ml
Cernevit 0,4 ml
Insulin 20 IU

For DNMP kidneys a total of 700 ml of perfusate was prepared and the first 200 ml of venous perfusate were discarded.

### NMP system

**Figure 2:**
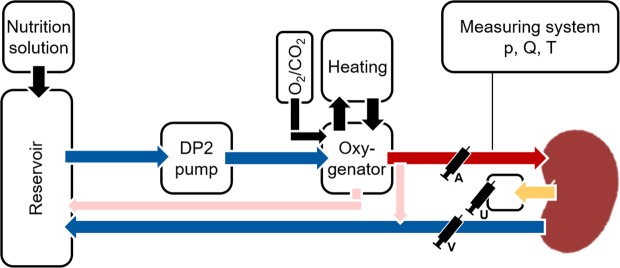
Normothermic perfusion system. The perfusate was pumped by a diagonal blood pump at a preset pressure of 75 mmHg from the reservoir through a heated and ventilated oxygenator into the renal artery and exited the renal vein into the reservoir. Pressure (p), flow (Q) and temperature (T) were measured continuously. Arterial (A), venous (V) and urine (U) samples were collected for subsequent analysis.

The perfusion medium in each circuit was circulated by a computer-controlled diagonal blood pump (Deltastream DP2, MEDOS Medizintechnik AG, Stolberg, Germany) and oxygenated using a heated (38 °C) oxygenator (Hilite 800, MEDOS Medizintechnik AG), ventilated with 1 l/min carbogen (95% O_2_/5% CO_2_; Linde AG, Leuna, Germany). From a venous reservoir (Capiox CR10NX, Terumo Deutschland GmbH, Eschborn, Germany), the medium was propelled by the pump through the oxygenator into the renal artery and exited the kidney through the renal vein into the reservoir (Fig. 2). During perfusion, a nutrition solution (Table 1) consisting of Nutriflex (B. Braun Melsungen AG) with Cernevit (Baxter Deutschland GmbH, Unterschleißheim, Germany) and Insulin (Sanofi-Aventis Deutschland GmbH, Frankfurt am Main, Germany) was added to each reservoir by a syringe pump (Cole-Parmer GmbH, Wertheim, Germany). A target mean arterial pressure (MAP) of 75 mmHg was maintained manually by adjusting a custom-designed pump controller (Informatik 11-Embedded Software, RWTH Aachen University, Aachen, Germany) and continuously monitored (DATEX AS/3, GE Healthcare; Solingen, Germany). Renal blood flow (RBF) was monitored via an ultrasonic flow sensor (SonoTT, em-tec GmbH, Finning, Germany). A temperature of 38 °C was maintained via a water bath thermostat connected to the oxygenators. Intrarenal resistance (IRR) was calculated as effectively measured MAP/RBF for each measurement.

### Perfusate and urine sampling and analysis

Samples of arterial and venous perfusate and urine were collected at distinct points in time (Fig. 1). Arterial and venous pO_2_ and pH levels were measured using a blood gas analyzer (ABL800 Flex, Radiometer GmbH, Krefeld, Germany). Renal metabolic activity was approximated by calculation of oxygen consumption ((c_a_O_2_ − c_v_O_2_)*RBF/kidney weight) by using arterial and venous oxygen contents, arterial and venous SO_2_ and pO_2_ values and hemoglobin concentrations (c_a/v_O_2_ = S_a/v_O_2_*1.34*c(Hb) + p_a/v_O_2_*0.0031). Urine was collected separately, and the urine output was recorded. The perfusate volume was replenished after measurement with basic Ringer’s solution to compensate for the excreted urine volume. Perfusate plasma samples and urine samples were stored at −80 °C for subsequent analysis. Perfusate samples were analyzed for sodium and creatinine levels. Protein, sodium and creatinine concentration were determined in urine samples. Using arterial perfusate and urine levels, creatinine clearance (urine creatinine*urinary flow/plasma creatinine/kidney weight) and fractional excretion of sodium (urinary sodium*plasma creatinine/plasma sodium/urinary creatinine) were calculated.

### Neutrophil gelatinase-associated lipocalin

Urinary levels of the acute tubular injury marker neutrophil gelatinase-associated lipocalin (NGAL, Kit 044, BioPorto Diagnostics, Gentofte, Denmark) were determined at the end of reperfusion using enzyme linked immunosorbent assay (ELISA) according to the manufacturer’s instructions. The absorbance was detected at 450 nm using a microplate reader (iMark^TM^, BioRadInc., Munich, Germany).

### Histology

After reperfusion, tissue samples from the renal cortex were fixed in 4% buffered formalin for 24 h and embedded in paraffin. Four-micron sections were stained with Hematoxylin Eosin (HE) and digitally scanned using an EVOS-microscope (EVOS-FL cell imaging system, Thermo Fisher scientific. Inc., Darmstadt, Germany). The histological images were analyzed according to a tubular injury score (0 = no injury, 1 = slight injury, 2 = prominent injury, 3 = necrotic cell injury), an osmotic nephropathy score (0 = no nephropathy, 1 = slightly nephropathy, 2 = strong nephropathy) and presence or absence of hypokalemic nephropathy. All analyses of the slides were performed by a senior nephropathologist in a blinded fashion.

### Statistical analysis

Statistical analysis was performed using GraphPad Prism 8 software package (GraphPad Software Inc., La Jolla, CA, USA). After performing a Shapiro-Wilk normality test, two-way analysis of variance (ANOVA) and multiple comparison were used followed by Bonferroni post-test correction for all measurements during perfusion. For kidney weight, hemorrhage and histological analysis, one-way ANOVA was applied. Data are presented as mean ± SEM and a p value < 0.05 was considered statistically significant.

## Results

### Kidney weight

**Figure 3:**
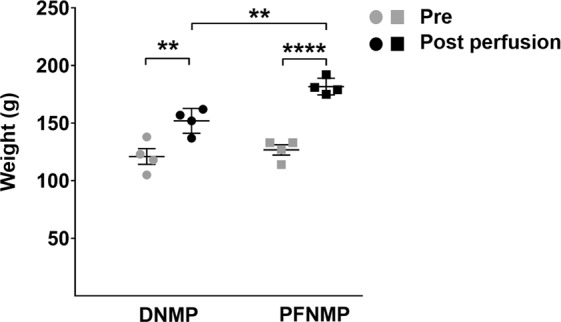
The kidney weight was comparable between the groups prior to the perfusion start (grey circles/boxes). After 6 h of perfusion, the weight was increased within both groups when compared to the starting weight, and significantly increased in the PFNMP group compared to the DNMP group (black circles/boxes). **p < 0.01/****p < 0.0001.

The weight of the kidneys was measured before and after reperfusion. Before reperfusion, the weights did not differ significantly between groups (121 ± 6.8 vs. 126.8 ± 4.5 g; DNMP vs. PFNMP respectively, Fig. 3). Both groups of grafts gained weight during 6 h of perfusion, however in the PFNMP group, the organ weight was higher in comparison to the DNMP group (181.8 ± 7.3 vs. 152 ± 10.8 g; p < 0.01, Fig. 3).

### Perfusion parameters

**Figure 4:**
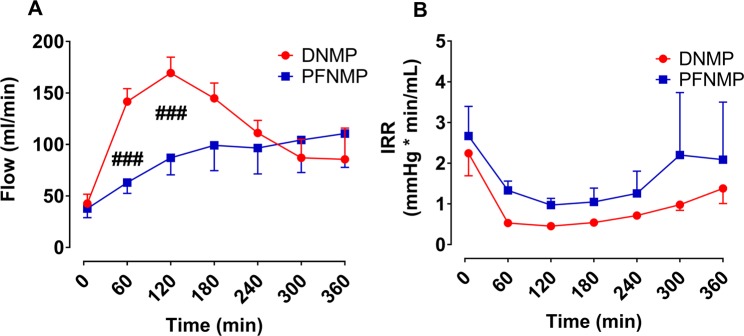
Renal blood flow was lower in the PFNMP group during the first two hours of perfusion (**A**), whereas the intrarenal resistance (IRR) did not significantly differ at any measurement (**B**). ^###^p < 0.001. (Short error bars of DNMP values from 60 to 240 min are hidden by symbols in **B**).

Renal blood flow was lower in the PFNMP group compared to the DNMP group at 1 and 2 h reperfusion (63 ± 4.7 vs. 141.6 ± 5.6 ml/min, p < 0.001 and 87 ± 7.4 vs. 169.4 ± 6.9 ml/min, p < 0.001 resp., Fig. 4A), while intrarenal resistance (IRR) showed a tendency to be higher in the PFNMP group during 6 h of reperfusion to the PFNMP group without reaching significance (Fig. 4B).

### Renal function

**Figure 5:**
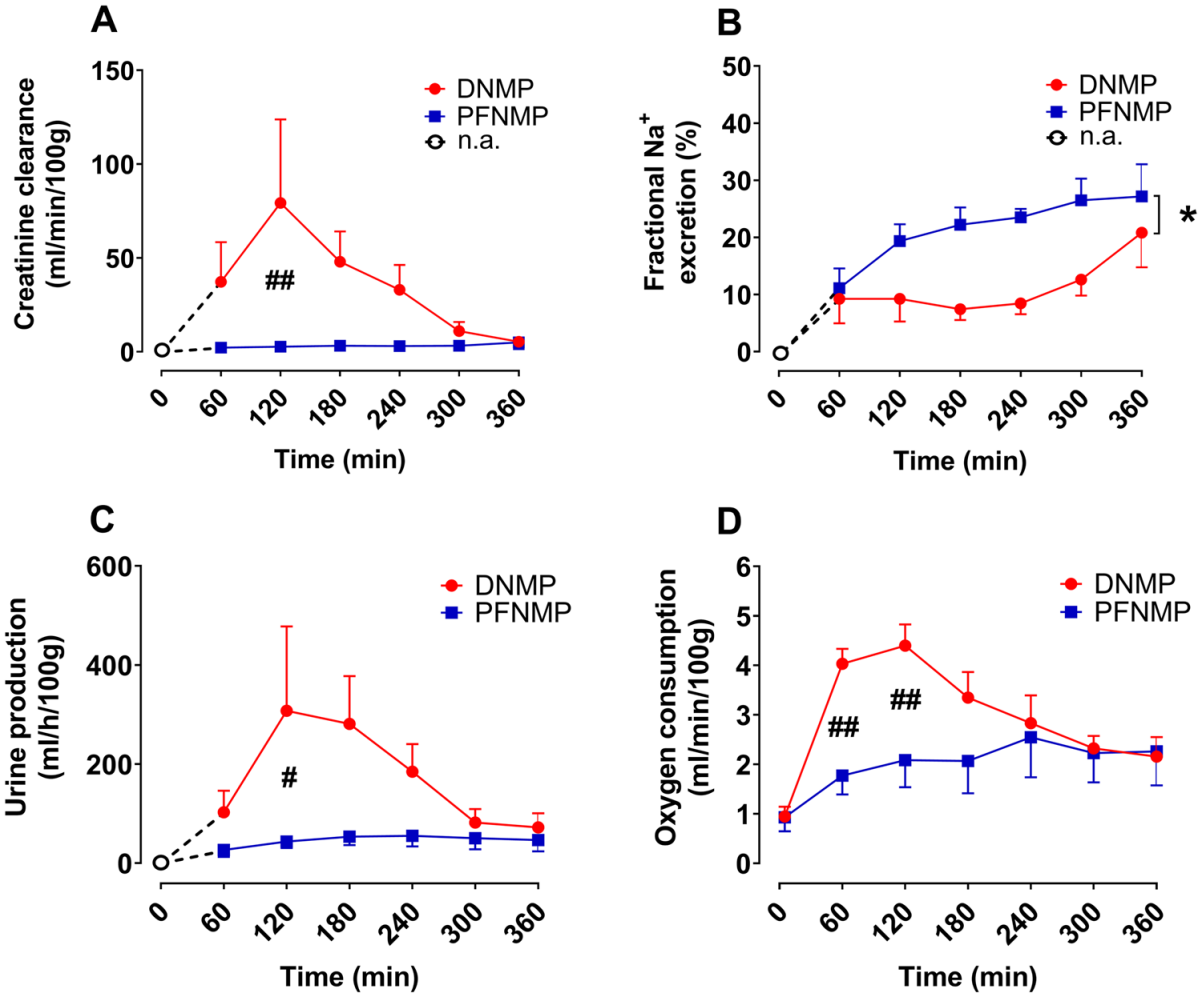
Creatinine clearance (**A**), urine production (**C**), and oxygen consumption (**D**) were lower in the PFNMP group compared to the DNMP group, whereas fractional sodium excretion was increased during 6 h NMP. (**B**) White circles and dotted lines indicate absence of urine production. *p < 0.05; ^#^p < 0.05/^##^p < 0.01.

Creatinine clearance was significantly lower in the PFNMP group at 2 h reperfusion and numerically lower in all measurements compared to the DNMP group (2.7 ± 0.4 vs. 79.3 ± 19.9 ml/min/100 g, p < 0.01, Fig. 5A). As a marker for tubular function, fractional excretion of sodium (FENa) was higher in the PFNMP group during 6 h reperfusion (p < 0.05 resp., Fig. 5B). Kidneys in the PFNMP group produced less urine compared to the DNMP group at 2 h (43.6 ± 5.8 vs. 307.6 ± 76.1 ml/min/100 g, p < 0.05, Fig. 5C) and showed a lower metabolic activity as expressed by oxygen consumption at 1 and 2 h reperfusion (1.8 ± 0.2 vs. 4.0 ± 0.1 ml/min/100 g, p < 0.01 and 2.1 ± 0.2 vs. 4.4 ± 0.2 ml/min/100 g, p < 0.01 resp., Fig. 5D).

**Figure 6:**
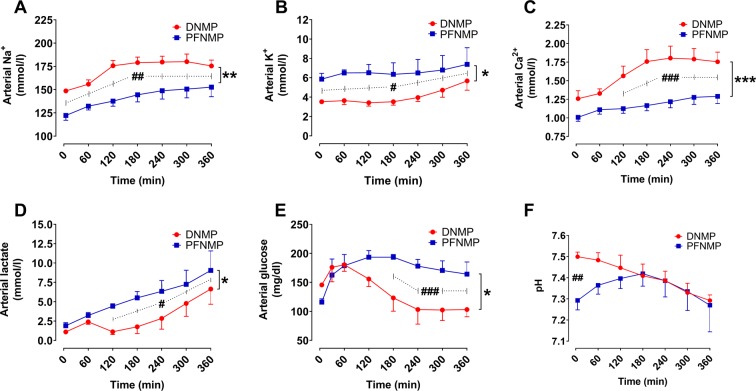
Arterial sodium (**A**) and calcium (**C**) concentrations were lower in the PFNMP group, whereas the arterial potassium (**B**), lactate (**D**) and glucose (**E**) concentrations were higher when compared to the DNMP group. (**F**) pH only differed at 5 min after perfusion start. *p < 0.05/**p < 0.01/***p < 0.001; ^#^p < 0.05/^##^p < 0.01/^###^p < 0.001.

The arterial concentrations of sodium were lower, while arterial potassium concentrations were higher in the PFNMP group at all measuring points (Fig. 6A, B). Arterial calcium and lactate concentrations were higher in the PFNMP group between 2 and 6 h (Fig. 6C, D). Glucose concentrations were higher during the perfusion period in the PFNMP group between 3 and 6 h (Fig. 6E). The pH differed between groups only after 5 min of perfusion with a lower pH in the PFNMP group (Fig. 6F).

**Figure 7:**
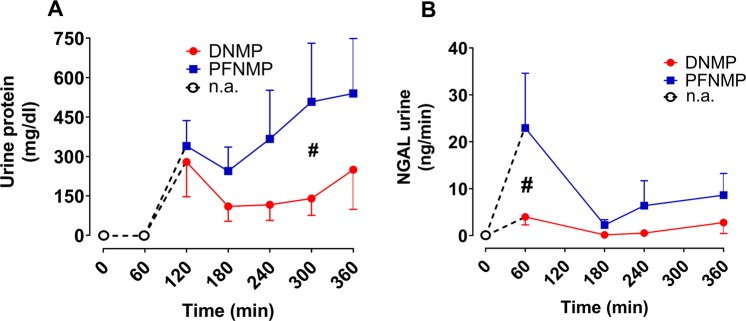
In the DNMP group, lower concentrations of urine protein (**A**) and a lower neutrophil gelatinase-associated lipocalin (NGAL) release in urine were seen at 5 and 1 h resp. White circles and dotted lines indicate absence of urine production. ^#^p < 0.05.

At 5 h reperfusion, the PFNMP group demonstrated higher urinary protein concentrations compared to DNMP (507.6 ± 99.7 vs. 140 ± 28.4 mg/dl, p < 0.05; Fig. 7A). Moreover, acute tubular injury as expressed by urinary NGAL levels was increased in the PFNMP group at 1 h NMP compared to the DNMP group (22.9 ± 11.7 vs. 3.9 ± 1.7 ng/ml, p < 0.05; Fig. 7B).

### Histology

**Figure 8:**
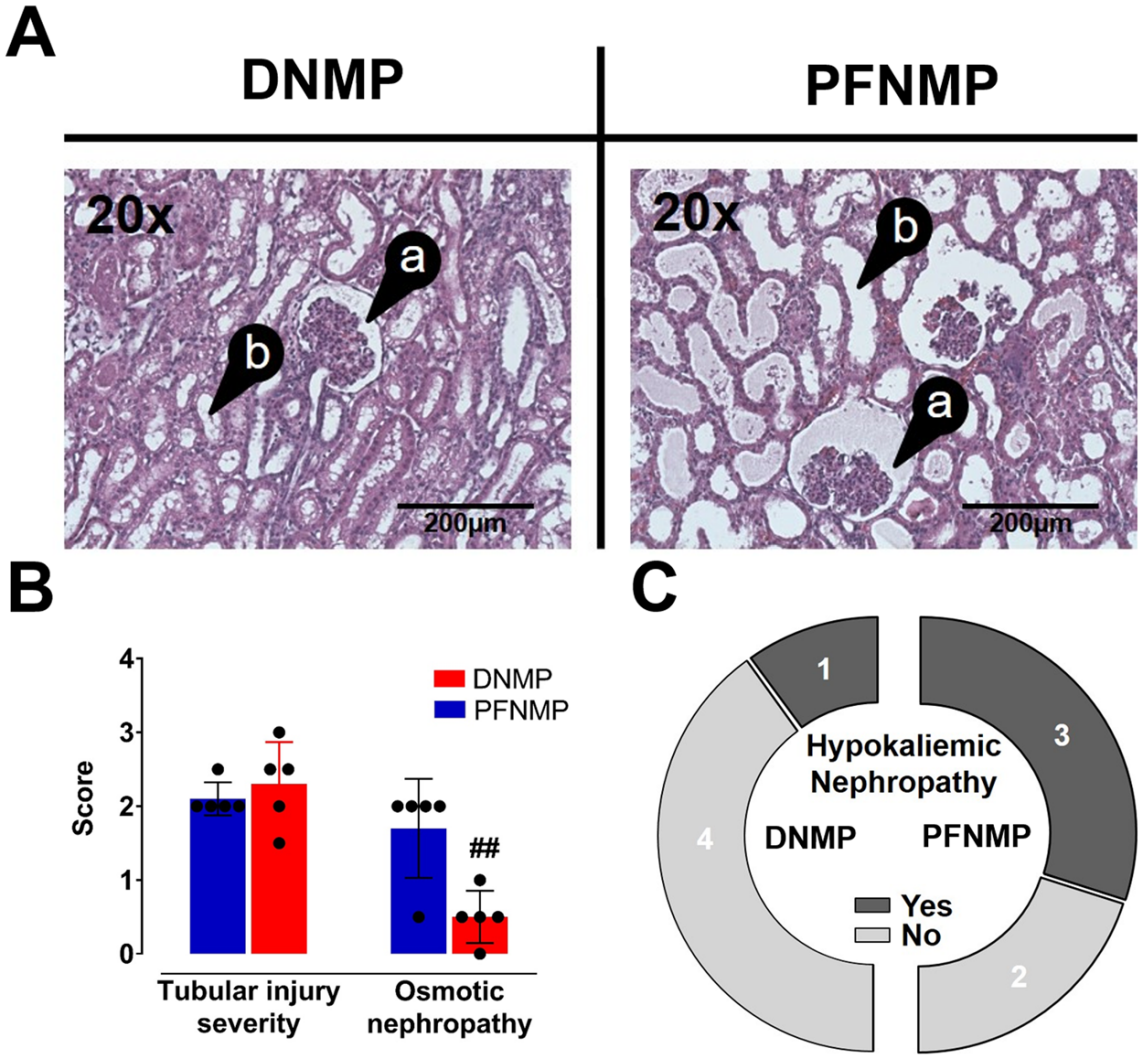
In the DNMP group, less dilation of the Bowman’s capsule (**A**/a) and tubular cell injury (**A**/b) was seen in comparison to the PFNMP group (Hematoxylin-Eosin stain, magnification x20), possibly related to significant higher osmotic nephropathy in the PFNMP group (**B**) and additionally, more prominent hypokalemic nephropathy in the PFNMP group (**C**). ^##^p < 0.01.

Microscopic examination of kidney cross sections after 6 h perfusion revealed diffuse acute tubular injury as the leading pathological finding, with tubular dilation, flattening and loss of brush border, consistent with ischemic type of injury (Fig. 8A), which showed a comparable grade between groups in its extent, i.e. in the area of affected parenchyma (Fig. 8B). Tubular cells necrosis was rare and only focal, but more often observed in the DNMP group. In the PFNMP group however, dilation of the Bowman’s capsules and anisometric vacuolization of the tubular cells, a sign of osmotic swelling (Fig. 8B) as well as tubular cells and tubular cross-sections with single large vacuoles, suggestive of hypokalemic nephropathy were increased in comparison to the DNMP group (Fig. 8C).

## Discussion

In this study, we investigated warm ischemia-damaged porcine kidneys, either preflushed with cold HTK solution (PFNMP group) or without preflush (DNMP group) prior to 6 h reperfusion using a normothermic machine perfusion (NMP) system. In comparison to the DNMP group, PFNMP kidneys gained more weight during reperfusion and histological samples showed a larger degree of osmotic nephropathy. Kidney function was impaired in the PFNMP group as indicated by deteriorated glomerular and tubular function, lower metabolic activity, higher lactate levels, higher concentrations of urine protein and a higher release of NGAL in urine. The detrimental effect of cold preflush was pronounced for approximately four hours after which the parameters in both groups converged.

NMP has been employed as a novel method for kidney preservation, in particular for resuscitation and viability assessment of warm ischemia-damaged grafts^2,3,5,8,9^. To our knowledge, in all reported NMP studies, kidneys were subjected to cold preflush. While a previous study reported increased histological injury after cold flush and cold storage followed by NMP^15^, our findings suggest that cold preflush prior to NMP has detrimental effects on kidney function and preservation quality, when compared to DNMP. Although the approach of direct NMP cannot be easily adapted into clinical practice as it is clinical standard to washout the donor’s vasculature with cold preservation solution before organ retrieval, avoiding or minimizing the detrimental effects should be studied further in the context of NMP. The employed preservation solutions might be optimized for use as flushing solution for NMP, since most preservation solutions were developed for optimal outcome in CS preservation. It has recently been shown that a combination of different solutions for flushing and CS can be superior to the use of a single solution^18^. Additionally, other approaches, such as warm flushing with a novel phosphate-free preservation solution (AQIX) prior to NMP^19^ and recently, normothermic regional perfusion as a method to reestablish circulation in DCD donors^20^, showed beneficial effects on kidney graft function and transplantation outcomes.

It has been demonstrated that HTK, UW, Celsior and Euro-Collins have comparable efficacy for flushing and cold storage preservation of kidney grafts^21^. We used HTK because it is the clinical standard solution for flushing and cold storage preservation of kidneys in Germany and has the largest market share of all preservation solutions worldwide. Moreover, for flushing of DCD kidneys HTK is also a frequently applied preservation solution due to the low viscosity, when compared to University of Wisconsin solution.

In the experiments, kidneys from young animals with a mean weight of 56 kg were procured and these were flushed out until the effluent was macroscopically free of blood remnants. We found in preliminary experiments that 200 ml of HTK was sufficient. Additionally, this is equal to the amount of perfusate that was discarded in the DNMP group.

During our study, after approximately 4 h of reperfusion, the beneficial effect in the DNMP group on functional parameters (renal blood flow, creatinine clearance, FENa, urine production and O2 consumption) returned to comparable levels seen in PFNMP. This might be on the one hand a result of hypokalemic nephropathy and severe hypernatremia, which were more pronounced in the DNMP group. An optimized perfusate composition for DNMP could potentially maintain the improved results in this group during the final two hours of reperfusion. On the other hand, another obvious explanation of the reduction of the beneficial effect of DNMP over time could be the rapid deterioration of kidney function in both groups found after approximately 3 h of NMP as expressed by IRR, creatinine clearance, FENa and urine protein. In contrast to NMP of other solid organs, studies of prolonged renal NMP are rare. A previous study using a similar Ringer’s-based perfusate for NMP of a porcine kidney with only 6.7 min of warm ischemia time reported also a decline in creatinine clearance after 3 h of perfusion time^22^. However, it was discussed by the authors that total elimination of the added creatinine was the cause.

Since continuous NMP has been shown to be superior to NMP combined with CS^3^, an extension of the achievable perfusion time could increase clinical application of NMP. A recent study showed that recirculation of urine could maintain electrolyte levels and achieve 24 h of NMP after CS^13^. Another group demonstrated 16 h of kidney perfusion time using a perfusion medium containing Steen solution, originally designed for lung NMP containing albumin and dextran^3^. In both experiments, oncotic pressure was maintained at a normal level by albumin, while our perfusate had an estimated sub-physiological oncotic pressure of 8.5 mmHg. In both groups of our study, a weight gain took place during perfusion, indicating edema and a potential contribution to the decline in kidney function over time. Unfortunately, the mentioned studies did not report kidney weights and gains. Additionally, another group reported that the use of leucocyte- and platelet-depleted blood in NMP results in improvement of renal function parameters^23^, indicating that an additional depletion of platelets might be beneficial. Aiming to extend achievable perfusion times and reduce graft injury, more research on optimal perfusion preparation methods and perfusion conditions is warranted, including systematic approaches to improvement of perfusion media since there are no comparative studies reported to date.

It could be argued that direct NMP was not applied, but rather a warm flush with perfusate which was thereafter discarded. However, cooling down of the kidney by cold flushing was avoided and the initial perfusion medium was discarded to eliminate accumulated hypoxia-induced and inflammatory mediators. In our study, for ethical reasons, kidneys were obtained from animals employed in other experiments. The kidneys, however, were procured after a comparable period of warm ischemia and before other procedures were performed on the animals.

The duration of NMP was limited to 6 h and the objective was to investigate the isolated effect of the cold preflush on organ grafts without influence from other organs. To assess, whether the observed detrimental short-term effects of cold flush lead to significant long-term effects and clinical outcomes such as primary non-function, delayed graft function and reduced graft survival, longer durations of NMP as well as a model using reperfusion with whole blood and eventually *in vivo* transplantation studies are necessary.

From this study, the mechanism of aggravated ischemia reperfusion injury in the PFNMP group remains unclear. The observed effect could be the result of the cold flush or subsequent rapid rewarming in the NMP system. A recent study compared cold-stored kidney grafts subjected to NMP with or without a controlled oxygenated rewarming (COR) procedure and found improvement of kidney function after COR^24^, suggesting that rewarming plays an important role, as evidenced also by the current study.

To conclude, the detrimental effect of cold flush prior to NMP was demonstrated when compared to direct NMP. Kidney function was impaired for the first 4 h of reperfusion and renal tissue damage at histological level was increased. Improved procedures for kidney preparation before NMP could potentially improve preservation quality and clinical outcomes. Moreover, further improvements to NMP are required to achieve prolonged perfusion durations necessary for routine clinical application of NMP for kidney grafts.

## Data Availability

The corresponding author declares that all raw data material is available at the Dept. of Anesthesiology, Medical Faculty, RWTH Aachen University in Aachen, Germany.
